# Elucidating the effects of heavy metals contamination on vital organ of fish and migratory birds found at fresh water ecosystem

**DOI:** 10.1016/j.heliyon.2023.e20968

**Published:** 2023-10-26

**Authors:** Bushra Nisar Khan, Hamid ullah, Yusra Ashfaq, Nageen Hussain, Usman Atique, Tariq Aziz, Metab Alharbi, Thamer H. Albekairi, Abdullah F. Alasmari

**Affiliations:** a Conservation Biolopgy Lab, Institute of Zoology University of the Punjab, Lahore, Pakistan; b Department of Zoology, Bahawalnagar Campus IUB, Bahawalpur, Pakistan; c Institute of Microbiology & Molecular Genetics, University of the Punjab, Pakistan; d Department of Biosciences and Biotechnology, College of Biological System, Chungnam National University, Daejeon, South Korea; e Department of Agriculture University of Ioannina, Arta, 47100, Greece; f Department of Pharmacology and Toxicology, College of Pharmacy, King Saud University, P.O. Box 2455, Riyadh, 11451, Saudi Arabia

**Keywords:** Mangla freshwater reservoir, Birds, Fish, Vital organs, Cadmium, Lead, Kidney, Brain

## Abstract

Heavy metal pollution in aquatic environments threatens marine life and lowers the quality of freshwater supplies. This research aimed to quantify the heavy metal concentration in fish, avian tissue, and water of the Mangla reservoir. Concentrations of heavy metals such as Cu, Cd, Cr, and Pb were determined in five vital organs of six species of fishes (*Cirrhinus cirrhosis, Catla catla, Hypophthalmichthys molitrix, Wallagu attu, Cyprinus carpio, Sperata seenghala*) and five species of migratory birds (*Anas strepera, Aythya ferina, Anas platyrhynchos, Anas crecca, Anas clypeata*) and water of Mangla dam. Heavy metal concentration was observed with AAS’ help after the samples’ complete chemical digestion. The average concentration of these metals in water samples was higher than the mean values in fish and birds. Heavy metals damaged fish and birds in the same order: Cd > Cu > Cr > Pb. The results of this research will hopefully prompt further consideration on the dangers of heavy metal contamination in freshwater ecosystem.

## 1 Introduction

One of the three main ecosystems on Earth, freshwater aquifers are essential to global ecology and the economy [1]. Water reservoirs are crucial for nutrient cycling and environmental preservation, but they are progressively being impacted by heavy metals, which may threaten their stability [[2], [3], [4]]. Heavy metals are highly hazardous to terrestrial and aquatic ecosystems and, therefore, to humans, birds, and aquatic life due to their toxicity, accumulative nature, and non-biodegradability [5]. Mangla Reservoir is an artificial and 12th largest freshwater reservoir in Pakistan. Mangla reservoir comprises five pockets, i.e., Mangla, Khad, Jari, Poonch, and Kannsi. It is linked to various wastewater systems to discharge the harmful toxins it contains. As a result, the water quality of the Mangla water reservoir is at risk. Disposing of industrial and domestic wastewater in the Mangla water reservoir is causing water quality poisonous heavy metals [6,7]. This metal pollution always poses a risk of adverse effects on fauna and flora of aquatic ecosystems and human health, water, soil quality, and crops, both directly and indirectly. Therefore, quantitative investigation of heavy metals in water ecosystems has become the focus of researchers worldwide [[8], [9], [10]]. The availability of metal to organisms in aquatic environments is determined by a variety of physicochemical parameters (pH, conductivity, ions, and so on) as well as biological factors. Among the biological variables influencing metal availability, the feeding behavior and habitat preferences of organisms have a significant influence [11]. These fundamental characteristics are influenced by physiological factors such as accumulation rates and binding capacity in an animal, as well as environmental influences such as temperature and feeding behaviors. As a result, the metal flux pathways in aquatic animals are dependent on specific properties of water chemistry, sediments, and the biological traits of the species [12].

Worldwide fish consumption has rapidly expanded in recent years, especially as its medicinal and nutritional benefits have come to light. Fish provide around 25 % of the animal protein consumed by humans [13]. Fish are a significant protein source enriched with critical vitamins, minerals, and unsaturated fatty acids [14]. To fulfill the daily need for omega-3 fatty acids, the American Heart Association (AHA) advises consuming seafood at least two times a week [15]. Fish consumption in developing nations is anticipated to increase by 57 % between 1997 and 2020, from 62.7 million tonnes to 98.6 million tonnes [[16], [17]]. However, heavy metals are regularly accumulated by fish from food, water, and sediments [18,19]. Metals can enter the body of fish through three different pathways: the body surface, the gills, and the alimentary canal. The Mangla reservoir acts as a nexus of ecological interactions, not only supporting a thriving fish population but also serving as an important stopover for migrating birds on their annual migrations. Fish populations in the reservoir are vital to its ecological integrity. They act as both predators and prey, contributing to the ecosystem's nutritional dynamics. The presence of heavy metals in the reservoir causes bioaccumulation of these contaminants in fish tissues [19]. This, in turn, may harm the health of fish populations. The reservoir also serves as a seasonal habitat for a variety of migratory birds, giving them access to food resources and a safe location to rest while traveling long distances [20]. The migratory birds visiting the freshwater reservoir also accumulate these metals in their bodies as the birds drink the contaminated water. On the other hand, birds also prey on fish that have high levels of heavy metals in their tissues, so toxins can build up in their bodies over time. This complex interaction highlights the intricate relationship between the reservoir's inhabitants and the potential cascade of effects caused by environmental pollution. Heavy metals are sometimes elevated at succeeding trophic levels of food chains, resulting in biomagnification of these compounds in the food chain. Bioaccumulation and biomagnification of toxic heavy metals in food chains have serious consequences for animal and human health [19]. Furthermore, the risk evaluation of these metals is an important issue, as eating food contaminated with toxic metals can lower essential nutrients and harm health [21]. The assessment of the metal concentration in fish can be expanded to consider possible adverse effects on health through risk analysis. This strategy often bases its decisions on the anticipated hazard quotient. (THQ). Additionally, the heavy metabolic status of fish is indicated by biometric tests like condition factor (CF). This study aims to discover the causes, pathways, and consequences of contamination of heavy metals in the food chain of a freshwater ecosystem. Also, the EDI and THQ for various fish species will be analyzed to ascertain the possible risks to human health. In addition, this research will help future scientists better understand the hazards posed by heavy metals to the aquatic system and the effects on human health.

## 2 Materials and methods

### 2.1 Study area

The Mangla water reservoir in Mirpur, Azad Kashmir, Pakistan, built on the Jehlum River, served as the study site. It is situated approximately 108 km southeast of Islamabad. The whole region covered by this water reservoir is 26,500 ha. The Mangla water reservoir is located between 33°00′ N to 35°12′ N and 73°07′ to 75°40′ E [21]. This water reservoir is divided into five pockets: Jari Kass, Mangla, Khad, Poonch, and Kannsi Pockets). The study of these five relied on water quality [22,23]. The Mangla reservoir serves as a central fish spawning area and feeding supply. It is home to 52 reported fish species [24], the abundance of which increases during seasons of high-water levels. Various freshwater fishes breed in the Mangla Dam [25].

Heavy metals were detected in water samples, fish, and migratory bird organs. Surveys of sample sites were done from November 2020 to January 2021.

### 2.2 Water samples

50 ml of water was sampled from each pocket, cleaned by storing them in 1 % nitric acid [8], and labeled in an aseptic container. Samples were delivered to the lab within 48 h of being collected for heavy metal analysis.

### 2.3 Organ samples of fish

**Fig. 1:**
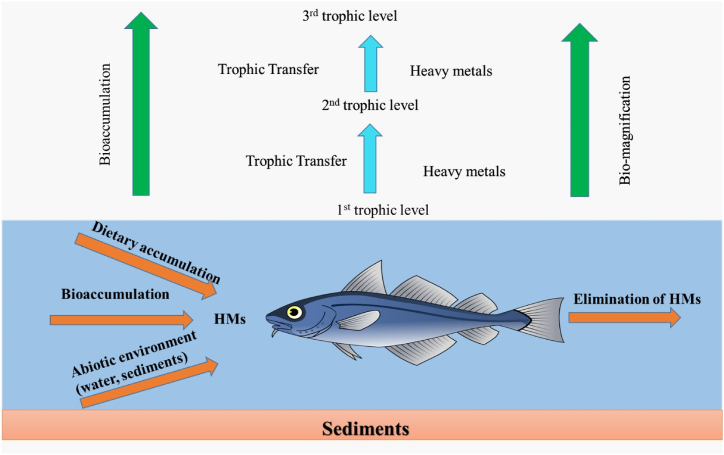
Schematic diagram outlining the keywords and ideas in the tropho-dynamics of heavy metals.

Annually the Wildlife and Fisheries Department, AJK leases out the fish of Mangla Dam to the fish contractor. These fish spots are very difficult to reach for collection of samples due to this reason 35 representatives of commercially important fish belonging to six species Mori (*Cirrhinus cirrhosis*), Thaila (*Catla catla*), Silver Carp (*Hypophthalmichthys molitrix*), Mali (*Wallagu attu*), Gulfam (*Cyprinus carpio*) and Singhara (*Sperata seenghala*) were collected in triplicate order with the help of fish contractor. Fish sampling was done at five sites namely Jari Kass, Mangla, Khad, Poonch, and Kannsi Pockets at the Mangla freshwater reservoir (Fig. 1). Five 30-m long, 1.5-m-deep gill nets, with stretch mesh sizes of 3 and 4 inches, were installed at these locations for experimental purposes. Different fish organs such as gills, liver, brain, kidney, and scales were taken from each fish sample. The desired samples were carried to the lab in an icebox for metal analysis [26].

### 2.4 Organ samples of birds

**Fig. 2:**
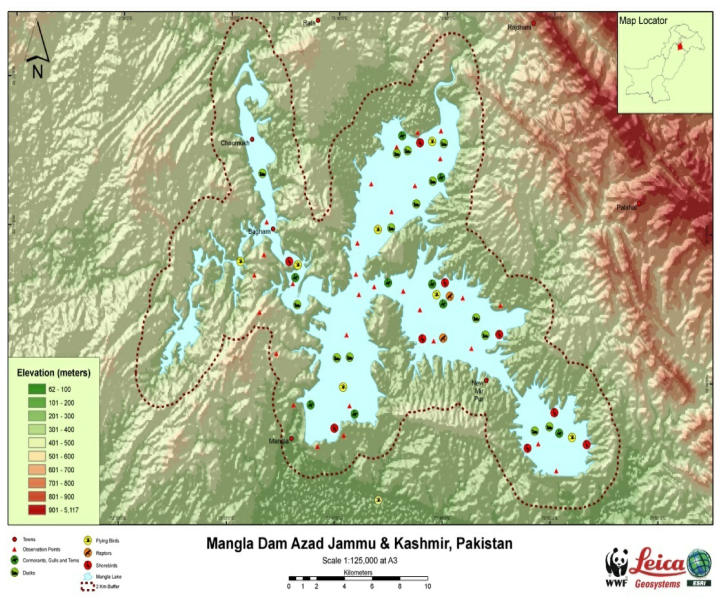
The five pockets of Mangla water reservoir: Jari Kass, Mangla, Khad, Poonch, and Kannsi Pockets.

In the current research work, the representatives of colonial migratory birds were selected for this purpose a total of 25 wild birds belonging to five species Gadwall (*Anas strepera strepera)*, Pochard (*Aythya ferina*), Mallard (*Anas platyrhynchos)*, Teal *(Anas crecca)* and Shoveler (*Anas clypeata)* were taken from each pocket Mangal dam shown in Fig. 2 were collected through simple point count methods with help of hunters [27,28]. At least five samples from each bird, including feathers, kidneys, liver, brain, and lungs, were taken aseptically in labeled plastic bags and transported and stored in the lab for further analysis.

### 2.5 Analysis of heavy metals

The fish samplings were thoroughly cleaned, dissected, and precisely weighed (wet weight) before being dried at 102 °C for 12 h. The specimens were considered again after cooling. Due to the advantages of this technology over traditional methods, such as shorter times, less acid use, and low containment in the solutions, microwave digesting processes have been used in many investigations in recent years [29]. The samples were digested following the methodology of Ahaneku & ADEOYE [30], and after digestion, samples were sent to the Environmental lab of the Botany department, Punjab University Lahore, for atomic absorption spectroscopy (Model: A Analyst 800, PerkinElmer).

### 2.6 Health risk assessment

#### 2.6.1 Coefficient of condition

Each fish sample's coefficient of condition (K) was estimated employing the procedure:

(Adams et al., [31]; Grimmett & Inskipp, [23]; WHO [32].

(1) $$\begin{array}{c} {}^{\circ}\;{}^{\circ}K=100\times \underline{W}\\ L\;3 \end{array}$$

L is the body length in centimeters, and W is the weight of the specimen in grams.

#### 2.6.2 Assessment of daily intake

The estimation of daily consumption resulting from the subsequent equation was used to determine whether or not consumers may be exposed to health risks from the researched metals [23,33].

(2) $$\begin{array}{c} {}^{\circ}{}^{\circ}ED\;I=\left(C\times IR\right)\\ BW \end{array}$$

Where IR denotes the consumption rate (in 250 g/day/person), BW denotes the weight of an adult human, and C represents the typical metal concentrations in muscle tissue (in μg/g, wet weight, 65 kg) [23,[33], [34], [35]].

#### 2.6.3 Target hazard quotient evaluation (THQ)

The possible non-carcinogenic risks to humans from pollutant exposure are evaluated using the target hazard quotient (THQ) [36,37]. The method for assessing THQ was provided by the USEPA Region III Risk-Based Concentration Table [38].

(3) $$\begin{array}{c} THQ=\left(C\;fish\times IR\times 10-3\times EFr\times EDtot\right)\\ \left(R\;f\;D\times BWa\times ATn\right){}^{\circ}\;{}^{\circ}\;{}^{\circ} \end{array}$$

EFr stands for the frequency of exposure (365 days), EDtot for total exposure (70 years), RfD for reference dose (μg/g/day), BWa for the weight of an adult (65 kg), and ATn for averaged time (EDtot 365 days/year) for non-carcinogens [23,39,40]. C fish stands for the average metal content in fish (g/g), and IR for the fish consumption rate (0.250 kg/day).

The hazard index (HI), which is equal to the sum of all metal hazard quotients, is determined using the target hazard quotient (THQ) for n-chosen metals [38].

(4) HI=THQ1+THQ2+...+THQn

THQ stands for the chosen n metals' target hazard quotients.

#### 2.6.4 Analytical statistics

Using Graphic PadPrism (version 5.03), a two-way analysis of variance (ANOVA) was conducted on the data to statistically analyze significant differences between the means of fishes and birds, heavy metals, and various species' essential organs. Two-tailed T-test (unpaired) was applied to compare metal concentrations in the organs of birds and fish.

## 3 Results

### 3.1 Physical parameters of water samples of the mangla reservoir

Different physical parameters of water such as pH, Chloride ions, Conductivity, COD, BOD, TDS, TSS, and Alkalinity were determined and found to be far higher than the standard by WHO given in (Table 1).

**Table: 1:** Physical parameter of water samples of the Mangla water reservoir.

Physical Parameters	Jarri Kass Pocket	Khad Pocket	Mangla Pocket	Poonch Pocket	Kannsi Pocket	Average	Standards (WHO, 2008)
pH	7.3	7.2	7.1	6.7	6.8	7.02 ± 0.26	6.5–8.5
Cl^−^ (mg/l)	112.01	96.11	79.02	20.31	42.16	69.8 ± 38.1	<250
Conductivity (μS/cm)	760.00	1750.09	1543.00	300.03	480.01	966.6 ± 646.1	1500
COD (mg/l)	40.00	120.10	184.02	24.01	50.00	74.6 ± 75.2	
BOD (mg/l)	23.02	11.00	19.01	17.03	30.08	14.6 ± 7.8	
TDS (mg/l)	471.00	1112.00	897.01	189.10	296.12	593 ± 396.3	<1000
Alkalinity (mg/l)	90.01	120.06	375.05	244.03	533.11	272.4 ± 184.2	200

### 3.2 Concentrations of heavy metals in water samples, determined in μg/L

It was noticed that overall average concentrations of heavy metals for five water sites were higher for Cd, followed by Pb, Cu, and Cr. However, an average concentration of all metals was found higher in Jarri Kass Pocket except Cu, which was recorded high at Khad Pocket. The trend of metal concentration in these pockets was Jarri Kass Pocket > Khad Pocket > Poonch Pocket > Kannsi Pocket > Mangla Pocket, as given in (Table 2).

**Table 2:** Heavy metal detection in water samples obtained from five pockets of Mangla water reservoir.

Sampling sites	Lead (μg/L)	Cadmium (μg/L)	Copper (μg/L)	Chromium (μg/L)
	Mean ± S.D			
Jarri Kass Pocket	18.62 ± 0.25	64.63 ± 0.70	7.42 ± 0.01	14.64 ± 0.01
Mangla Pocket	7.83 ± 0.02	13.94 ± 0.02	8.02 ± 0.04	10.43 ± 0.03
Khad Pocket	9.73 ± 0.01	10.44 ± 0.07	20.24 ± 0.05	9.04 ± 0.008
Poonch Pocket	12.65 ± 0.03	10.62 ± 0.07	15.26 ± 0.02	9.93 ± 0.08
Kannsi Pocket	10.92 ± 0.02	11.64 ± 0.02	7.34 ± 0.4	8.42 ± 0.005
Average	11.95 ± 1.8	22.25 ± 10.6	11.65 ± 2.61	10.49 ± 1.09
DWSP^1^	≤0.05	0.01	2	≤0.05
WHO (Edition, 2011)	10	3	2	5

DWSP^1^: Drinking water standard for Pakistan.

### 3.3 Concentrations of heavy metals in specific organs from several fish and avian species

#### 3.3.1 Lead

**Fig. 3:**
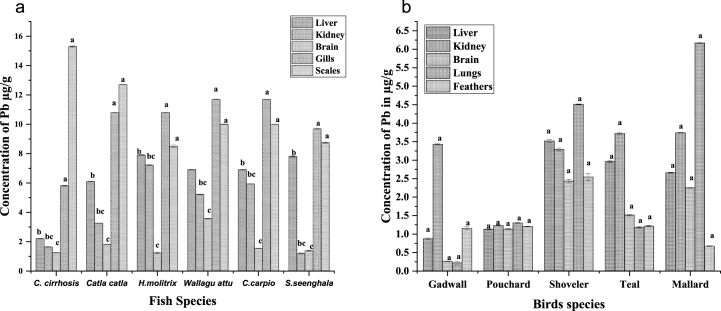
(a, b) The overall Pb concentrations in several fish and bird species' organs. Significant changes between means within each period indicate different letters (P 0.05, Tukey's HSD, ANOVAs). For each therapy, the bars show the means and SE.

Compared to bird organs, the amounts of Pb in fish organs were often higher. In fish, the highest concentration of lead was observed in sales of *C. cirrhosis* (15.3 ± 0.04 μg/g), and the lowest was in the brain of *H.molitrix* (1.23 ± 0.02 μg/g). While in the case of birds, Shoveler's lungs were shown to have the most significant levels of Pb (4.51 ± 0.008 μg/g), and the lowest was in the brain of Gadwall (0.226 ± 0.008 μg/g). The trend of Pb concentrations in fish has also been observed in *C. cirrhosis > C. catla > S.seenghala > W.attu > C.carpio > H.molitrix* (Fig. 3 a). The trend of Pb concentrations in birds was also observed in Shoveler > Mallard > Teal > Pouchard > Gadwall, as shown in (Fig. 3b).

#### 3.3.2 Cadmium

**Fig. 4:**
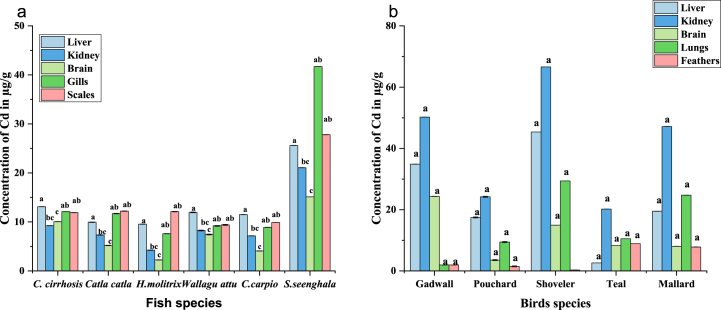
(a, b) The overall Cd concentrations in the organs of fish and bird species. Significant changes between means within each period indicate different letters (P 0.05, Tukey's HSD, ANOVAs). For each therapy, the bars show the means and SE.

Cd concentration in birds’ organs was higher than that of fish. In fish, the highest concentration of Cd was observed in sales of *S.seenghala* (41.7 ± 0.13 μg/g), and the lowest was in the brain of *H.molitrix* (2.25 ± 0.01 μg/g). In birds, the highest concentration of Cd was observed in the Kidney of the Shoveler (66.6 ± 0.01 μg/g), and the lowest was in the feathers of the Pouchard (1.5 ± 0.05 μg/g). The trend of Cd concentrations in fish has also been observed in *S.seenghala > C. cirrhosis > C. catla > W.attu > C.carpio > H.molitrix* (Fig. 4a). Concentration pattern of Cd results Shoveler > Gadwall > Mallard > Teal > Pouchard as shown in (Fig. 4b).

#### 3.3.3 Copper

**Fig. 5:**
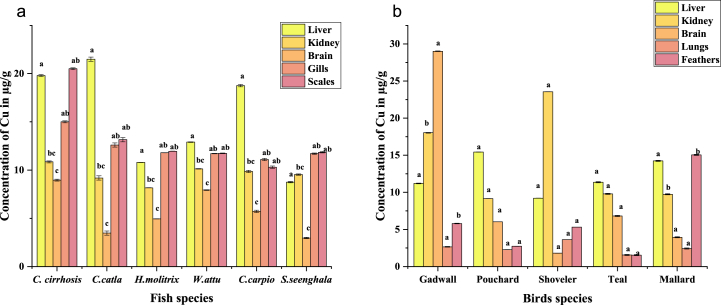
(a, b): The overall Cu concentrations in the organs of fish and bird species. Significant changes between means within each period indicate different letters (P 0.05, Tukey's HSD, ANOVAs). For each therapy, the bars show the means and SE.

It was noticed that overall concentrations of Cu in the organs of fish were higher than that of birds. In fish, the highest concentration of Cu was observed in the liver of *C. catla* (21.5 ± 0.22 μg/g), and the lowest was in the brain of *S.seenghala* (2.96 ± 0.01 μg/g). In birds, the highest concentration of Cu was observed in the kidney of Shoveler (23.56 ± 0.12 μg/g), and the lowest was in the lungs of Teal (1.56 ± 0.65 μg/g). Furthermore, the organ-wise trend was also observed in both fish and birds. In fish, the most affected organ was the liver, scales, gills, kidneys, and brain. The most affected organ in the case of birds was the kidney, followed by the liver, brain, feathers, and lungs. The trend of Cu concentrations in fish has also been observed in *C. catla > C. cirrhosis > C.carpio > W.attu > H.molitrix > S.seenghala* (Fig. 5a). The trend of Cu concentrations in birds was also observed Shoveler > Gadwall > Pouchard > Mallard > Teal as shown in (Fig. 5b).

#### 3.3.4 Chromium

**Fig. 6:**
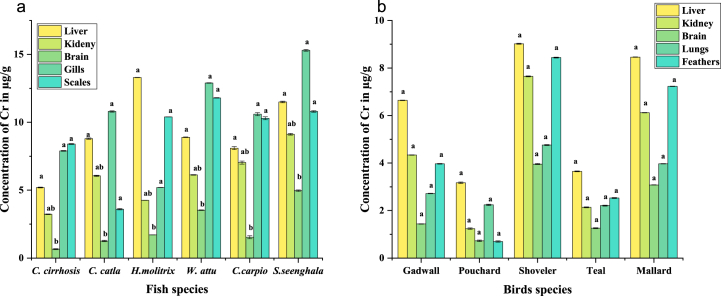
(a, b) The overall Cr concentrations in the organs of fish and bird species. Significant changes between means within each period indicate different letters (P 0.05, Tukey's HSD, ANOVAs). For each therapy, the bars show the means and SE.

It was noticed that overall concentrations of Cr in fish organs were higher than that of birds. In fish, the highest concentration of Cr was observed in the gills of *S.seenghala* (15.3 ± 0.11 μg/g), and the lowest was in the brain of *C. cirrhosis* (0.66 ± 0.02 μg/g). In birds, the highest concentration of Cr was observed in the scales of Shoveler (10.24 ± 0.008 μg/g), and the lowest was in the liver of Gadwall (0.24 ± 0.001 μg/g). Furthermore, the organ-wise trend was also observed in both fish and birds. In fish, the most affected organ was the gills, followed by the liver, scales, kidney, and brain. The most affected organ in the case of birds was the liver, followed by the kidney, feathers, gills, and brain. The trend of Cr concentrations in fish has also been observed in *S.seenghala* > *H.molitrix* > *W.attu* > *C. catla* > *C.carpio* > *C. cirrhosis* (Fig. 6 a). The trend of Cr concentrations in birds was also observed Shoveler > Pouchard > Mallard > Teal > Gadwall as shown in (Fig. 6 b).

##### 3.3.4.1 Comparison of heavy metals concentration in organs of birds and fish

**Fig. 7:**
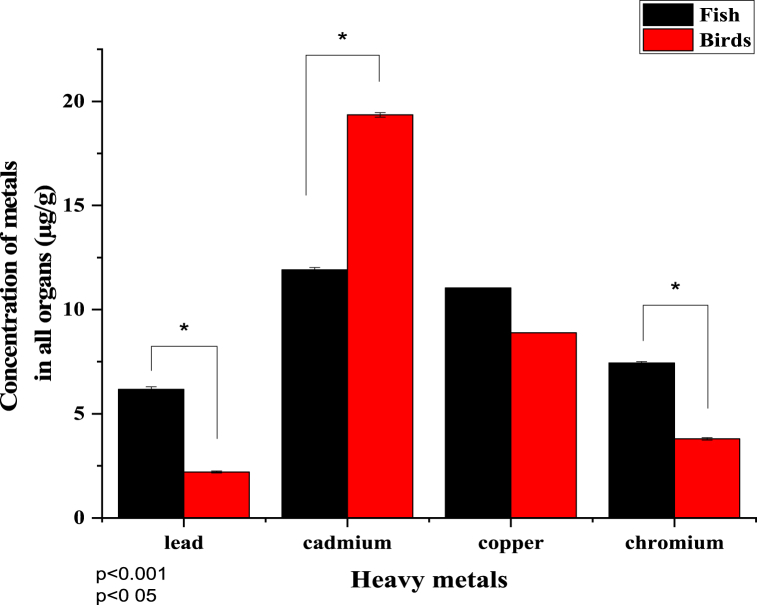
Average metal concentration in different organs of fish and birds. Asterisk (*) indicates a significant difference between the mean of metal concentration among the species. The obtained means of both species for each metal were compared using a *t*-test (p < 0.05).

All organs' overall average metal concentration was also compared (Fig. 7). Heavy metals' concentration in fish and birds' organs was compared using a two-tailed *t*-test with a significant difference in P values (P < 0.05) except for Cr and Cu concentrations in the organs of birds and fish. It was noticed that in this study, cadmium concentration was higher, and lead concentration was lower.

#### 3.3.5 Correlation between physicochemical parameters of water and heavy metals in water, fish and birds

The correlation between various water parameters and heavy metals in water, fish, and birds is displayed in Table 3. In water, Pb and Cd were positively correlated with pH chloride ions and BOD and negatively correlated with COD and TDS, while copper negatively correlated with pH chloride ions and BOD and positively correlated with COD and TDS. Cr was positively correlated with chloride ions and BOD and negatively correlated with COD and TDS. When it comes to fish, Pb, Cd, Cu, and Cr, all have positive correlations with BOD and negative correlations with pH, chloride ions, COD, and TDS. For birds, Pb, Cd, Cu, and Cr had positive correlations with pH, chloride ions, and total dissolved solids and negative correlations with biological oxygen demand (BOD).

**Table 3:** Pearson Correlation coefficient (r) between physicochemical parameters of water and different heavy metals in water, fish, and birds.

LABEL		PH	CL^−^ (mg/l)	COD (mg/l)	BOD (mg/l)	TSDS(mg/l)
Water	Pb	0.22287	0.23388	−0.60677	0.40242	−0.55167
	Cd	0.63612	0.65249	−0.35224	0.21443	−0.13179
	Cu	−0.22449	−0.28599	0.53895	−0.2575	0.11504
	Cr	0.66172	0.66048	−0.33769	−0.07723	−0.02906
Physico-chemical	pH	1	0.9969	0.41538	−0.28684	0.67808
	Cl- (mg/l)		1	0.38555	−0.22924	0.6562
	COD (mg/l)			1	−0.3775	0.83536
	BOD (mg/l)				1	−0.63339
	TDS (mg/l)					1
Fish	Pb	−0.74265	−0.70374	−0.87891	0.53819	−0.88197
	Cd	−0.52563	−0.49748	−0.98956	0.43496	−0.90132
	Cu	−0.09083	−0.04669	−0.92618	0.46613	−0.68482
	Cr	−0.43704	−0.40648	−0.99715	0.34592	−0.81358
Physico-chemical	pH	1	0.99748	0.23111	−0.16834	0.54048
	Cl- (mg/l)		1	0.20147	−0.11506	0.51711
	COD (mg/l)			1	−0.40939	0.83264
	BOD (mg/l)				1	−0.64225
	TDS (mg/l)					1
Birds	Pb	0.21134	0.17979	−0.2021	−0.80606	0.25514
	Cd	0.65261	0.64316	0.11455	−0.70785	0.6344
	Cu	0.94978	0.95631	0.46116	−0.34707	0.79283
	Cr	0.58997	0.61393	−0.48542	0.01144	−0.05437
Physico-chemical	pH	1	0.9969	0.41538	−0.28684	0.67808
	Cl- (mg/l)		1	0.38555	−0.22924	0.6562
	COD (mg/l)			1	−0.3775	0.83536
	BOD (mg/l)				1	−0.63339
	TDS (mg/l)					1

### 3.4 Health risk evaluation

#### 3.4.1 Fish biometric information

For several fish species, biometric data (length, weight, and coefficients of condition: K) were calculated. The mean levels for W, L, and K were 3483 g, 69.66 cm, and 1.04, respectively. (Table 4).

**Table 4:** Statistical summary of biometric data for various Mangla water reservoir fish species.

	Weight (g) (W)	Length (cm^3^) (L)	Coefficient of condition (g/cm^3^) (K)
Min	3000	65	0.72
Max	4000	76	1.33
Mean	3483	69.66	1.04
SD	371.0	4.50	0.25
SEM	151.4	1.83	0.10

#### 3.4.2 Target hazard quotient (THQ) assessment

The estimated daily intake (EDI), target hazard quotient (THQ), and hazard index are used to quantify the heavy metal risk. (HI). The risk assessments of different fish species for human health were also examined, and the outcomes are summarized in Table 5.

● PTDI, which stands for permissible tolerated daily intake (mg/day/70 kg body weight),
● Estimated daily intake (EDI, mg per day per 70 kg of body weight)
● Target Hazard Q factor
● *WHO/FAO (FAO, 1983; Organization, 2002)

**Table 5:** Lists the health dangers connected to particular metals identified in the flesh of different fish species from the Mangle water reservoir.

Metals	RfD (μg g^−1^ day^−1^)	PTDI	*C. cirrhosis*		*C. catla*		*H. molitrix*		*W. attu*		*C. carpio*		*S. seenghala*	
			EDI	THQ	EDI	THQ	EDI	THQ	EDI	THQ	EDI	THQ	EDI	THQ
Lead	0.004	0.250	14.44	0.66	14.81	0.75	27.35	1.11	17.53	0.91	43.62	4.71	21.31	1.38
Cadmium	0.001*	0.070	41.00	12.83	41.00	13.00	47.00	18.00	47.00	19.00	49.00	20.00	46.00	16.00
Copper	0.04*	35	54.86	1.18	105.00	1.00	43.00	0.73	60.00	1.00	43.00	0.73	17.5	0.41
Chromium	0.003*	0.210	22.00	0.30	27.00	0.47	32.00	0.47	26.00	0.45	30.00	0.55	30.00	0.18
Hazard Index (HI)				3.75		3.80		5.00		5.34		6.49		4.49

## 4 Discussion

**Fig. 8:**
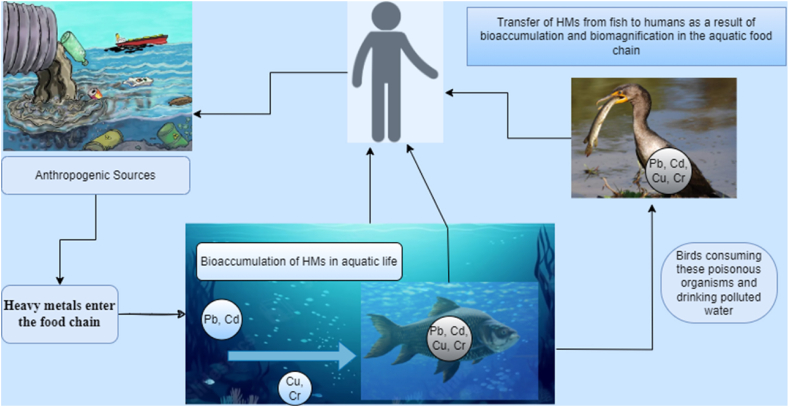
Schematic diagram of the human food chain regarding bio-accumulation of heavy metals. The HMs, with time, accumulate in bottom sediments, enter the food chain and affect aquatic life, birds, and humans.

Heavy metal toxicity is rapidly contaminating water in developing nations like Pakistan. Industrial and domestic wastes, shipping, and landfill runoff introduce these metals to wetlands and coastal waters. Metals in bottom sediments penetrate the food chain and harm aquatic life, birds, and humans (Fig. 8). Agriculture, employment, and environmental standards have improved because of the Mangla water reservoir. These benefits are enhanced by additional water and power generation. With high flow, several streams pollute reservoirs [41]. Mangla water has heavy metals like cadmium, lead, chromium, and copper.

In the present study, an analysis of the physical and chemical parameters of water and heavy metals in water, different fish, and bird organs of Mangla Lake was carried out. Physical water parameters, such as pH, Chloride ions, Conductivity, COD, BOD, TDS, TSS, and Alkalinity, were determined. It was noticed that the range (6.7–7.3) of pH values of five individual sites and the average value (7.02) of entire places was below the recommended levels of WHO [42]. (Table: 1). The levels of pH in the current study were comparable to Ref. [43]. However, drinking a pH above 8.0 was not recommended [44]. The level of Mangla water alkalinity showed that the water was characteristic of alkaline nature, and the dissolved carbonates largely prevailed as HCO3 [45]. Our study showed that the pH of the Mangla water was within limits prescribed by international authorities [44]. pH has significant impacts as a higher pH level of water may decrease the solubility of Cd, Cu, and Pb. When compared to water with a high pH, which can breakdown metal complexes and releases gratis metallic ions into the water [46]. Mean BOD levels in Mangla water were recorded as high as given in another study of Mangla Mangla water reservoir [7]. The dissolved oxygen levels were lower than those measured in Pandoh Lake, Bharat [47] and Lake Beysehir, Turkey [48]. Variability of BOD often concerns flow regimes, seasonal effects, and impacts caused by anthropogenic activities [49]. Our study results varied in TDS and Conductivity as a range of total dissolved substances 189–1112 mg/l, while Electrical Conductivity ranged from 300 to 1750 S/cm. These values were higher than those found in other studies on Pandoh Lake [50], Mangla Dam, Pakistan [8], and even more significant than limits set by alternative bodies [44]. It directed that the water from the Mangla water reservoir had elevated salinity and mineral content [51]. The of Mangla Dam were measured during the winter season and the physicochemical concentrations are related to seasons. As the season changes there is a fluctuation in the physicochemical characteristics of the water and aqautic food chain. This may be due to the change in inflow and temperature due to seasonal variations. Resultantly physicochemical parameters of the freshwater ecosystem influence the bioavailability of heavy metals in this way there was a correlation between physicochemical properties on the content of heavy metals in water, fish and birds as given in Table 4 [52]. As per Pearson Correlation coefficient the chloride ions and biological oxygen demand (BOD) were positively linked with pH for Pb and Cd in water. In fish, Pb, Cd, Cu, and Cr correlate positively with BOD and negatively with pH, chloride ions, COD, and total dissolved solids. Biological oxygen demand (BOD) was negatively correlated with Pb, Cd, Cu, and Cr in birds, while pH, chloride ions, and total dissolved solids all had positive relationships.

The levels of Chloride ions were measured to be 69.6 mg/l in the present study. The measured Cl− levels were higher than those in Manchar Lake [53] and other studies [8,52]. The most probable sources of Chloride ions in Mangla Lake may be the deposition of atmospheric pollutants, waste effluents, physical, biological, and chemical weathering of rocks, and agricultural runoff. Water with higher concentrations of Chloride ions is also unsafe for drinking and aquatic fauna [54].

However, the average concentration of all metals was higher in Jari Kass Pocket except Cu, which was recorded as high in Khad Pocket. The trend of metal concentration in these pockets was Jari Kass Pocket > Khad Pocket > Poonch Pocket > Kannsi Pocket > Mangla Pocket, as given in (Table 2). The highest concentrations of all metals, especially at Jari Kass Pocket, are due to 80 % automobile workshop wastes, municipal wastes, oil spilling from the boat for traveling and fishing, and agricultural runoff. The results for Cd, Cr, and Pb concentrations in our study were meager compared to those reported for the Karnaphuli River in Bangladesh [54]. The Cd, Cr, and Pb concentration was compared with various standards used by Ref. [54], and it was noticed that Cd amounts were above the recommended limits, but Cr and Pb were more or less comparable. Moreover, a study regarding the water quality of Mangla Lake was conducted in different seasons by Saleem et al. [8], that also reports alarming amounts of Cd, Cr and Pb, Ni, and Co recorded in all seasons in this lake. The higher concentration of these metals in water may be because their attention is higher in sediments compared to the surrounding water [55]. In aquatic environments, metals are present in trace amounts. The outcome is heavy metal contamination in bodies of water since these metals cannot dissolve and are therefore deposited and integrated into water, sediments, and aquatic animals [56,57]. The study also recommended immediate action to conserve the healthy aquatic ecosystem of this lake.

Heavy metals from sediments penetrate aquatic vegetation, which fish and birds eat. Heavy metal solubility, absorption, and precipitation affect fish and avian heavy metal accumulation (Fig. 1). Heavy metals also enter the birds when they feed on fish and aquatic plants of polluted Mangla Lake. Heavy metals, including Pb, Cu, Cd, and Cr, accumulate in tough and delicate tissues, including the liver, kidney, brain, feathers, and scales [58].

Lead is relatively insoluble. The gastrointestinal tract absorbs a small amount of it. Lead poisoning occurs after intake in the host. It then travels to the liver, kidney, brain, and blood. Fish had more concentration of information than birds. An organ-wise trend was observed in fish and birds. (Fig. 3a, (b)). In fish, the most affected organ was the scales, followed by gills, liver, kidney, and brain. A study reported a higher amount in liver and gills of *C. carpio* and Pelteobagrus fluvidraco fishes collected from Meiliang Bay, Taihu Lake, China. Still, our findings for Cr and Pb were controversial in their results [59]. Pb concentrations exceeded the permissible limit of 2.0 μg/g as described by the Punjab pure food rules, 2011. The mean Pb level was more significant than the acceptable international limit [[60], [61], [62]], indicating a potential threat to people who consume Catla catla. The trend of Pb concentrations in fish has also been observed *in C. cirrhosis > C. catla > S.seenghala > W.attu > C.carpio > H.molitrix*.

In the bird's case, the most elevated lead concentration was observed in the kidney, while the lowest concentration was in the brain. In the study we conducted, Pb exposure values in the liver and kidneys of birds were lower than those mentioned by Sara et al. [63]; Scheuhammer, [64]. More than five μg/g dw of Pb in the brains of birds indicates poisoning, while levels above sixteen μg/g dw indicate extensive metal contamination [64,65]. None of the Pb levels in the brains in our study went above the hazardous limit. In our study, none of these birds exceeded the threshold limit of metals in feathers reported by Dahmardeh Behrooz et al., [65]. The birds eat primarily fish, while crabs and mollusks may be consumed when fish density or quantity is low in their feeding areas [66]. Fish, mollusks, and crustaceans have higher metal contents in their bodies, according to research [67,68]. Moreover, excessive Pb accumulation in birds has been connected to an increase in cellular stress brought on by the metabolic inhibition of proteins, fats, and carbohydrates [69]. The study emphasized the memory loss in birds described as a result of increasing Pb levels in water birds [70]. The lead absorption in different organs of the fish and bird are (p < 0.0001) found to be significant. Coal, mineral fertilizers, and household garbage release cadmium into the air. Cadmium causes cancer and mutagenesis [71]. Cadmium poisoning can kill over time. Cadmium poisoning damages the kidneys the most [72]. It is a highly toxic metal that is mainly consumed through contaminated food [73,74].

In our study, cadmium levels in fish and birds were the highest of the other heavy metals. It could be because of the greater concentration of Cd in the water. In fish, the highest concentration of Cd was observed in sales of *S.seenghala*, and the lowest was in the brain of *H.molitrix*. The least amount of Cd was reported in organs of *Cyprinus carpio* and *Hypophthalmichthys molitrix* that was sold in the market of district Lower Dir, Khyber Pakhtunkhwa, Pakistan, as they reared in freshwater as compared to our study [75]. In comparison, a higher concentration of silver carp was reported due to the Olifants River, the most polluted river in South Africa [76]. Concentrations of Cd and Cr in all fishes exceeded the permissible limit of two μg/g and four μg/g according to available standards of WHO and FDA. For Cd, scales have higher amounts, followed by gills, liver, kidney, and brain, while the concentration in all organs was lower than the average concentration of Cd in water. Furthermore, the organ-wise trend was also observed in both fish and birds (Fig. 4 (a), (b)).

The highest value of cadmium was observed in kidneys, and the lowest value was observed in feathers. Our study's results on Cd exposure levels in the liver and kidney were higher than the values suggested by Scheuhammer [64], which were three and eight μg/g dw, respectively. These values indicate toxicities, and according to Bakshi & Panigrahi [77], Cd concentrations in the liver and kidney above forty μg/g dw and 100 μg/g dw indicate hazardous exposure [78]. The cadmium threshold level in avian feathers is two μg/g [65]. In our study, teal and mallard feathers had the most excellent cadmium level. Any other birds did not exceed this limit. The most affected organ in the case of birds was the kidney, followed by the liver, lungs, brain, and feathers. Concentration pattern of Cd results Shoveler > Gadwall > Mallard > Teal > Pouchard. The Cd absorption in different organs of the fish and bird are (p < 0.0001) found to be significant. Although copper (Cu) is essential to several enzymes for creating hemoglobin and bone density, excessive ingestion can harm the liver and kidneys [74,79]. Anthropogenic sources of copper in soil include mining, waste discharges, and using sewage, fungicides, and fertilizers in agriculture [80]. Cadmium can enter a wetland through natural and anthropogenic pathways; however, the vast majority of cadmium in surface waters comes from anthropogenic sources Water pollution at Mangla Dam was caused primarily by weathered rock, industrial waste, and pesticides. Because the birds being studied are omnivores, their diets do not exclusively consist of fish. Cd accumulation in birds may also increase when there is a higher concentration of Cd on the water's surface. According to Figure-7, which displays the average metal concentration in different organs of fish and birds from all studies, migratory birds had a higher concentration of Cd (19.35 mg/g) than fishes. However, fishes have higher concentrations of all other harmful metals. Fig. 4 (a & b) shows unequivocally that bird populations have a greater Cd concentration than fish populations. Assimilation rates are reliant on the chemical characteristics of every metal and the tissues in which they are retained. The higher accumulation of cadmium in the kidneys of birds may be justified by the high vascularization of kidney tissues, the long half-life of the metal, and the slow removal from the tissues [81,82]. The findings of Garcfa-Fernanndez et al. [83], were similar to the current study as they suggested that the kidney is the predominant organ for accumulation of cadmium in bird samples, followed by the liver and, to a lesser extent, the brain and bone. Mariko et al. [84] also found a higher concentration of Cd in the livers of 85 wild birds. The Cd builds up to dangerous levels in the kidneys of birds; the highest concentration of Cd in the kidneys of migrating ducks was also observed by Binkowski et al., 2015, who studied the region around the Zator River in Poland [85].

It was observed that overall concentrations of Cu in the organs of fish were higher than that of birds (Fig. 5 (a), (b)). Cu was discovered in the current investigation in all the examined fish samples, with an average value of 11.04 μg/g. More considerable than previously reported, the moderate Cu content of fish tissues was found (66–69]. Compared to our study, Cyprinus Carpio and *Hypophthalmichthys molitrix*, raised in freshwater, had the lowest Cd, Cu, and Pb levels in their muscles when sold in the lower-class district market in Khyber Pakhtunkhwa, Pakistan [75]. The concentration of Cu in waterbird organs and feathers has been reported to be lower in various parts of the world [71,72]. The most affected organ of birds in our study was the kidney, followed by the liver, brain, feathers, and lungs. The trend of Cu concentrations in birds was also observed Shoveler > Gadwall > Pouchard > Mallard > Teal. The Cu absorption in different organs of the fish and bird are (p < 0.0001) found to be significant. A crucial micronutrient for fish and birds is chromium. One of the anthropogenic sources of Cr in the environment is its usage in the chemical, obstinate, and metallurgical sectors.

Generally, birds are excellent indicators of chromium. Affected areas of birds due to Cr include feathers, liver, kidney, heart, and skin [74]. Chromium toxicity relates to alteration in hematological parameters in birds and directly affects the blood and blood-related mechanisms. A biochemical change was also observed in recent research that proves chromium's destructive effects on birds, which indirectly alters the chromium levels in the human body when we consume these toxic birds [73]. The threshold level of chromium in birds is 2.8 μg/g [74], but the current study showed a higher level in waterbirds that might harm them [75]. According to a survey, dietary preferences may contribute to greater Cr exposure in waterbirds [76]. A higher level of Cr was found in fishes [55], and these waterbirds mainly feed on fish, which could explain why the Cr was higher in the two waterbird species investigated. Fish had an average Cr content of 7.44 μg/g. All samples contained chromium; the highest concentration was 10.45 μg/g, above the WHO [42] guideline of 0.15 μg/g and the USPEA [[86], [87], [88], [89]] threshold of Eight μg/g.

These findings agreed that Cr concentration in fish tissues was enhanced due to biomagnification at each trophic level and mainly concentrated in carnivorous bottom dwellers [77]. In another study, changes in water pH were also documented as an essential cause for the accumulation of Cr in fish tissues [78]. Cr concentrations remain higher in gills than in other body organs, even at the same pH [77]. In fish, the most affected organ was the gills, followed by the liver, scales, kidney, and brain. The trend of Cr concentrations in all fish has also been observed *S.seenghala* > *H.molitrix* > *W.attu* > *Catla catla* > *C.carpio* > *C. cirrhosis*. The Cr absorption in different organs of the fish and bird are (p < 0.0001) found to be significant. Except for Cr and Cu, a two-tailed *t*-test showed significant differences in heavy metal concentrations in fish and bird organs (P < 0.05). The kidney has higher metal contents than the brain. Gills > liver > scales > kidney > brain was the fish organ metal trend. Organ-wise, birds have kidneys > livers > lungs > feathers> and brains. The average metal content in all organs was compared. This study found higher cadmium and lower lead concentrations. Cd,>Cu > Cr > Pb was the average metal concentration trend in birds and fish. (Fig. 7).

Eating fish may have both cancer-causing and non-cancerous impacts and significant health risks. Heavy metals may be present in water reservoirs in small amounts, but certain kinds of fish may gather these metals through biological accumulation and biomagnification. As a result, people who eat fish often may develop high amounts of pollutants in their bodies [18]. The daily intake was estimated to assess the level of exposure from fish consumption. Pb, Cu, Cd, and Cr all had EDI levels more incredible than the recommended PTDI values (Table 5). Freshwater fish from Pakistan's Mangla Dam should receive particular concern due to the more significant amounts of Pb, Cd, Cr, and Cu in the flesh of the fish. The THQ method evaluated the non-carcinogenic hazards to people who ingest several fish species from Mangla Dam. THQ readings above 1 suggest that consuming contaminated fish may pose a non-carcinogenic health risk to consumers [[90], [91], [92]]. Eating contaminated fish may provide certain non-carcinogenic risks because the Cd, Cr, Cu, and Pb measured THQ values were higher than unity. The lifelong non-carcinogenic health concerns for consumers were indicated by the highest HI value of 6.49. Nevertheless, commercially important fishes were selected and sold in this sequence: *Wallagu attu, Catla catla, Cirrhinus cirrhosis, Hypophthalmichthys molitrix, Cyprinus carpio, Sperata seenghala*. According to heavy metal analysis, these fish have all assessed heavy metal concentrations over permitted limits in their bodies (Table 2). Even so, *Hypophthalmichthys molitrix* was the safest fish to eat. Recent studies show that the water reservoirs' metal removals are rising; stricter regulations are needed to prevent further damage to aquatic ecosystems.

## 5 Conclusion

We conclude that both natural and human-caused pollution impact local aquatic biodiversity. This study demonstrates that aquatic species, fish, and birds living in polluted areas consume toxins that build up in their tissues. Heavy metals in water were not having the same effect on all fish and bird species, and there were no discernible patterns of metal accumulation across the body. Our research indicates that the dietary requirement was less necessary than the number of heavy metals found in fish from the Mangla water reservoir, a common food source for the local population and locations. Due to their inherent toxicity, heavy metals pose complex health problems once they enter the human metabolic system. Reducing these concentrations in drinkable water is urgently needed to prevent serious health problems.

## Data availability statement

Data will be made available on request.

## CRediT authorship contribution statement

**Bushra Nisar Khan:** Methodology, Conceptualization. **Hamid ullah:** Formal analysis. **Yusra Ashfaq:** Methodology. **Nageen Hussain:** Investigation. **Usman Atique:** Conceptualization. **Tariq Aziz:** Supervision, Resources, Funding acquisition. **Metab Alharbi:** Data curation. **Thamer H. Albekairi:** Writing – review & editing. **Abdullah F. Alasmari:** Investigation.

## Declaration of competing interest

The authors declare no conflict of interest.
